# Malignant glomus tumor of prostate: A case report

**DOI:** 10.3389/fonc.2023.1121307

**Published:** 2023-03-30

**Authors:** Zhanxin Sun, Fuzhen Sun, Chunhong Yu, Helong Xiao, Qingle Xu, Bo Gao, Liuxiong Guo, Junjiang Liu, Shoubin Li

**Affiliations:** ^1^ Graduate School of Hebei North University, Zhangjiakou, Hebei, China; ^2^ Department of Urology, Hebei General Hospital, Shijiazhuang, Hebei, China; ^3^ Department of Health Examination Center, Hebei General Hospital, Shijiazhuang, Hebei, China

**Keywords:** prostate, glomus tumor, malignant, urinary tract, diagnosis

## Abstract

We reported an 85-year-old patient with malignant glomus tumor (GT) of the prostate. He presented with urinary frequency for more than 2 years and gross hematuria for 7 days. Computed tomography scan showed that the prostate was markedly irregularly enlarged, and the boundary between the prostate and the posterior wall of the bladder was unclear. Bilateral kidneys and ureters were dilated. Biochemical examinations showed that the serum potassium was 7.24 mmol/L and the serum creatinine was 974.6 μmol/L. Transurethral diagnostic resection was performed after restoring homeostasis through several times of bedside blood filtration. The pathological diagnosis was malignant GT. The patient’s renal function recovered after bilateral nephrostomy, and he refused further treatment and was out of contact after 9 months. We summarize the clinical and histopathological features of malignant GT of the prostate in order to improve the early recognition of the disease by clinicians.

## 1 Introduction

Glomus tumors (GTs) are mesenchymal neoplasms consisting of a combination of glomus cells originating from normal glomus, blood vessels, and smooth muscle cells (1). Most GTs are benign and often occur in the skin of the head and neck, muscles, and distal limbs, especially the nail bed (2). Malignant GTs are extremely rare accounting for <1% of all GTs. Only a few cases of malignant GTs have been reported, and most of them were locally aggressive and distally metastatic (3).

In this study, we report a case of aggressive GT probably originating from the prostate. A systematic literature review was performed using PubMed (MEDLINE) for searching. There are no published reports of malignant GTs invading the prostate, and this may be the first case. This report extends the differential diagnosis of prostate tumors.

## 2 Case presentation

**Figure 1:**
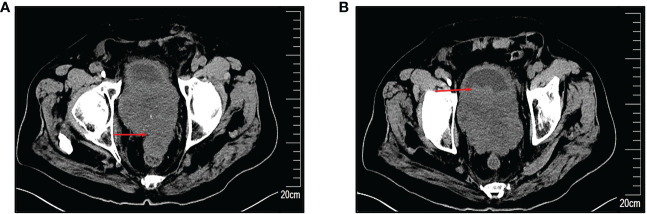
Preoperative computed tomography (CT) scans. **(A)** Markedly enlarged prostate with irregular morphology, measuring approximately 58×93×106mm, with an indistinct border with the rectum. **(B)** Indistinct demarcation of the prostate from the posterior bladder wall. The red arrow indicates the site of the lesion.

An 85-year-old man was admitted on 2 January 2022 for a chief complaint of urinary frequency for more than 2 years and gross hematuria for 7 days. The patient had a history of urinary frequency for 2 years but was not under regular treatment or medication. He had no history of bladder or prostate cancer and no history of any chronic medical conditions. Physical examination revealed edema in bilateral lower limbs. Biochemical tests showed high serum potassium (7.24 mmol/L), blood urea nitrogen (92.67 mmol/L), and serum creatinine (974.6 μmol/L). His serum prostate-specific antigen (PSA) level was in normal range (1.66 ng/ml). Bilateral hydronephrosis and a low-equal echo mass behind the bladder approximately 60×93×65mm in size were found in ultrasound examination. There was irregular enlargement of prostate, measuring about 58×93×106mm, with uneven internal echo. Computed tomography (CT) scan showed that the bladder wall was thickened, especially in the posterior wall. The prostate was irregular in shape, significantly enlarged in volume and with indistinct boundaries to the rectum. The local border between the prostate and the posterior wall of the bladder was poorly delineated (**Figures 1A, B**). After several times of bedside hemodialysis, the patient’s hyperkalemia and renal failure gradually recovered.

**Figure 2:**
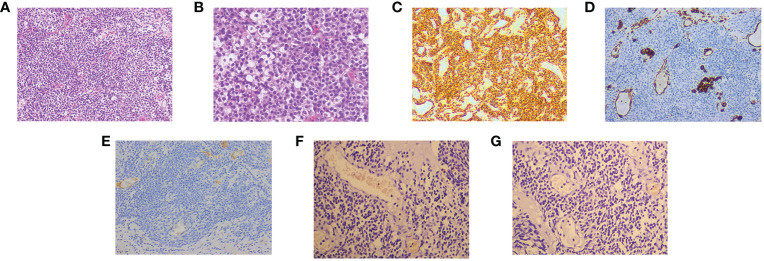
The hematoxylin–eosin (HE) and immunohistochemical pictures. **(A)** The tumor cells are uniform in size, round in shape, well-defined, and distributed around blood vessels (HE, original magnification, 100×). **(B)** The tumor cells show diffuse heterogeneous proliferation, increased mitotic signs, and visible multifocal necrosis (HE, original magnification, 200×). Immunohistochemistry: SMA **(C)**, Vimentin **(D)**, Desmin **(E)**, TFE3 **(F)**, and HMB45 **(G)**.

**Figure 3:**
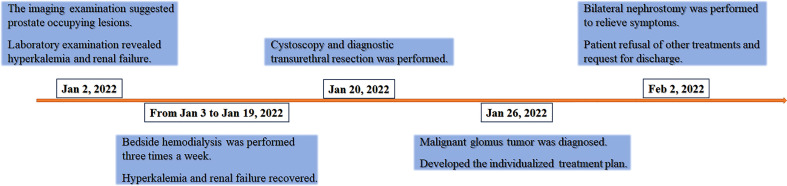
Treatment timeline of the malignant glomus tumor case.

Cystoscopy and transurethral diagnostic resection were performed on 20 January 2022. The prostate gland was found to be significantly enlarged and protruded into the bladder under cystoscopy. There were multitudinous cauliflower-like masses measuring approximately 3.0×4.5cm in extent in the portion of the prostate that protruded into the bladder and the bladder neck. Bilateral ureteral orifices were not visible. Part of the prostate that protruded into the bladder was excised for pathological examination. Histopathology examination revealed that the tumor cells were oval and uniform in size. The cells had a clear boundary and were mainly distributed around the blood vessels. Tumor cells showed diffuse atypia with increased mitotic activity and multifocal necrosis under high power field (**Figures 2A, B**). Immunohistochemical markers were diffusely positive for smooth muscle actin (SMA), while they were negative for vimentin, HMB45, TFE3, and desmin (**Figures 2C–G**). These findings were highly suggestive of a malignant glomus tumor. Considering the clinical stage and the patient’s physical condition, combined with the financial situation, the patient and his family refused to receive additional treatment. The patient was discharged with bilateral nephrostomy and was required to recheck regularly. The patient failed to show for follow-up after 9 months. The treatment timeline for this patient is summarized in **Figure 3**.

## 3 Discussion and conclusion

The diagnosis of malignancy should take into account tumor size, infiltration, mitotic activity, nuclear atypia, and vascular involvement (4). By summarizing the pathological features of 52 cases, Folpe and colleagues proposed a subclassification of atypical and malignant GTs (5). The criteria for the diagnosis of malignancy are as follows: i) deep location of the tumor, ii) size of the tumor>2cm, iii) atypical mitosis or obvious nuclear heterogeneity, iv) mitotic cells accounting for five or more of 50 under high power field (HPF). Malignant GTs have a high rate of distant metastasis. The overall metastasis rate is 38% for cutaneous malignant GTs. Common metastatic sites are the bone, brain, liver, lung, small intestine, and adjacent lymph nodes (6, 7). If a GT is larger than 2cm and deep in location, but without nuclear heterogeneity, it will be classified as an undetermined malignant potential GT (8).

GTs rarely originate from the urinary tract. Only six cases of GT occurring in the bladder have been reported up to now (9, 10). Gross hematuria is often the only symptom of GT in the lower urinary tract. Radiographic examination can evaluate the tumor’s location, size, depth of infiltration, and distant metastasis. In the case of malignant GT of the bladder, distant metastases can occur at early stage (11). It is difficult to diagnose a malignant GT by radiographic imaging. The accurate diagnosis relies on pathological examination. The ectopic GTs have the same histological and immunohistochemical features as those in limbs; it is vital for the diagnosis of ectopic GTs (12). In immunohistochemistry of GTs, the marker for smooth muscle actin (α-SMA) and muscle-specific actin (MSA) are mostly positive, while the epithelial markers (e.g., CK5/6, CK7, CK20, and CKpan), CD56, CD34, CD31, and EMA are negative (9, 13–15).

In this case, the giant tumor of the prostate had invaded the posterior wall of the bladder, and its pathological features meet the diagnostic criteria of malignant GTs. Considering the rigorousness, it needs to be differentiated from the following diseases. First is prostate cancer, which mostly originated from the peripheral zone of the prostate gland and where the serum PSA is elevated and the immunohistochemical markers such as P50, PSA, and PAP are positive. Second is the prostate lymphoma (PL), which is rarely observed, too, and mostly occurs in relatively young patients. Heterogeneous lymphocytes arranged in sheets can be seen under the microscope, and immunohistochemical markers of lymphocyte origin are positive (16). Third is the small cell prostate neuroendocrine carcinoma (SCPC), which is another rare and highly aggressive malignant tumor. The tumor cells are small and round or short spindle-shaped and are focally positive for chromogranin A, CD56, and synaptophysin in immunohistochemistry and have a high Ki-67 proliferation index (17). This tumor lacks the expression of androgen receptors and does not secrete PSA (18). Fourth are the rhabdomyosarcoma tumors, which are characterized by soft tissue differentiation features. Tumor cells have abundant cytoplasm and positive expression of mesenchymal-derived immunohistochemical markers (19). Fifth are the perivascular epithelioid neoplasia (PEComas), which is also mesenchymal in origin, and the malignant ones have similar histological features to malignant GT (20). The immunohistochemical markers are usually strongly positive for HMB45, SMA, and TFE3 (21), but in this case, immunohistochemistry was negative for HMB45 and TFE3. Sixth is the bladder neck tumor. Cystoscopy showed that the prostate was protruded into the bladder. We resected part of the prostate tissue that protruded into the bladder and sent it for pathological examination; combined with the opinion of our MDT, the tumor was considered to have primary origin in the prostate.

There are no definite treatment protocols for malignant GTs of the prostate at present. We can refer to the treatment of GTs that occurred in other organs to make the therapy plan. Transurethral resection of bladder tumor (TURBt) is an effective method for benign (22) or undetermined malignant potential (23) GTs of the bladder. Malignant GTs are highly aggressive with poor prognosis. Shim et al. treated a patient with malignant GT of the bladder with a combination of TURBt and chemotherapy, whereas the patient died of pulmonary failure due to tumor metastasis 2 months after surgery (11). The optimal treatment of malignant GT remains undefined, and the current published literature only consists of individual case reports or series. In this case, the tumor had invaded the bladder; systemic and local therapy may be beneficial to some extent. We offered the patient the therapy options of radical prostatectomy combined with post-operative radiotherapy and chemotherapy, but the patient and his family refused additional treatment due to financial reasons. Further examination such as chest CT and bone scan can help us to clarify the clinical stage, confirm the presence of distant metastasis, and develop an individualized treatment plan. 18F-Fluorodeoxyglucose FDG PET/CT can help us to identify the primary site of a tumor. However, due to financial reasons, the patient and his family refused further examinations. Pathological examination of a portion of the prostate tissue sample obtained by diagnostic TURP and normal prostate tissue could not be identified in the tissue specimen provided. Thus, the tissue sample obtained by this method was abnormal, and the examination method needs to be improved and further examination performed to obtain a more accurate tissue condition. Thus, we did not have enough evidence to be certain that the tumor was considered to have primary origin in the prostate, which is the limitation of this case report.

In summary, this report reminds us that in case of irregular enlargement of the prostate with a normal serum PSA level, the possibility of malignant glomus tumor should be considered. For GT of the prostate, the clinical manifestations are non-specific, and pathological examination is the gold standard of diagnosis. It is important to involve multidisciplinary teams in the management of the disease. For early confined lesions in the prostate, radical prostatectomy is the preferred surgical procedure. For the cases in advanced clinical stage, the appropriate treatment scheme needs further study.

## Data availability statement

The original contributions presented in the study are included in the article. Further inquiries can be directed to the corresponding authors.

## Ethics statement

Ethical review and approval was not required for the study on human participants in accordance with the local legislation and institutional requirements. The patients/participants provided their written informed consent to participate in this study. Written informed consent was obtained from the relatives of patient for the publication of any potentially identifiable images or data included in this article.

## Author contributions

ZS, SL, and FS obtained and analyzed the clinical data. ZS, CY, and HX wrote the manuscript. QX, BG, and LG designed and constructed the figures. JL and SL designed and supervised the study and edited the manuscript. All authors contributed to the article and approved the submitted version.
